# Mechanism Enhancing Arabidopsis Resistance to Cadmium: The Role of *NRT1.5* and Proton Pump

**DOI:** 10.3389/fpls.2018.01892

**Published:** 2018-12-19

**Authors:** Tao Wang, Yingpeng Hua, Moxian Chen, Jianhua Zhang, Chunyun Guan, Zhenhua Zhang

**Affiliations:** ^1^Southern Regional Collaborative Innovation Center for Grain and Oil Crops in China, College of Resources and Environmental Sciences, Hunan Agricultural University, Changsha, China; ^2^Hunan Provincial Key Laboratory of Farmland Pollution Control and Agricultural Resources Use, Hunan Provincial Key Laboratory of Nutrition in Common University, National Engineering Laboratory on Soil and Fertilizer Resources Efficient Utilization, Changsha, China; ^3^Department of Biology, Hong Kong Baptist University and State Key Laboratory of Agrobiotechnology, The Chinese University of Hong Kong, Shatin, Hong Kong; ^4^Department of Biology, Hong Kong Baptist University, Hong Kong, China; ^5^National Center of Oilseed Crops Improvement, Hunan Branch, Changsha, China

**Keywords:** ABA signaling, *NRT1.5*, *NRT1.8*, NO_3_^−^, Cd stress, proton pump activity

## Abstract

**Aim:** Heavy metal pollution is serious in China, and abscisic acid (ABA) is an important stress hormone. How it regulates plant tolerance to cadmium remains unclear, so we aimed to explore the molecular mechanism responsible for enhanced cadmium resistance in Arabidopsis wild-type and mutant plants and *Brassica napus* seedlings.

**Methods:** Arabidopsis/*B. napus* were cultured hydroponically for 28/15 days and then treated with 20/10 μM Cd/Cd+ABA (5 μM) for 3/4 days. Chlorophyll degradation rate, SPAD values, proline, MDA, ABA, ${\mathrm{NO}}_{3}^{-}$, and Cd concentrations were measured in root vacuoles and protoplasts; root to shoot ${\mathrm{NO}}_{3}^{-}$ and Cd concentration ratios were determined and *NRT1.5-, NRT1.8-, BnNRT1.5-*, and *BnNRT1.8-*related gene expression was studied.

**Results:** Cytoplasmic ABA levels in root cells of *bglu10* and *bglu18* Arabidopsis mutants were significantly lower than those in the wild-type, apparently making the latter more resistant to Cd. ${\mathrm{NO}}_{3}^{-}$ long-distance transporter *NRT1.5* responded to ABA signaling by downregulating its own expression, while *NRT1.8* did not respond. Concomitantly, proton pump activity in wild-type plants was higher than in the *bglu10* and *bglu18* mutants; thus, more ${\mathrm{NO}}_{3}^{-}$ and Cd accumulated in the vacuoles of wild-type root cells. ABA application inhibited Cd absorption by *B. napus*. *BnNRT1.5* responded to exogenous ABA signal by downregulating its own expression, while the lack of response by *BnNRT1.8* resulted in increased amount of ${\mathrm{NO}}_{3}^{-}$ accumulating in the roots to participate in the anti-cadmium reaction.

**Conclusion:** *NRT1.5* responds to the ABA signal to inhibit its own expression, whereas unresponsiveness of *NRT1.8* causes accumulation of ${\mathrm{NO}}_{3}^{-}$ in the roots; thus, enhancing Cd resistance. In Arabidopsis, because of proton pump action, more ${\mathrm{NO}}_{3}^{-}$ and Cd accumulate in the vacuoles of Arabidopsis root cells, thereby reducing damage by Cd toxicity. However, in *B. napus*, the addition of exogenous ABA inhibited Cd absorption. Our data provide a sound basis to the theoretical molecular mechanism involved in hormone signaling during response of plants to heavy metal stress.

## Introduction

Nitrogen (N) is an essential macronutrient that plays a key role in plant growth and development, and in crop yield (Hirel et al., 2007; Wang et al., 2012; Krapp et al., 2014; Ruffel et al., 2014; Vidal et al., 2014). Nitrates (${\mathrm{NO}}_{3}^{-}$) are some of the most abundant N sources in natural and agricultural systems (von Wiren et al., 2000). Absorption, transport, sensing, and responses to ${\mathrm{NO}}_{3}^{-}$ have been extensively studied (Krapp, 2015; O’Brien et al., 2016). In addition to its role as a nutrient ${\mathrm{NO}}_{3}^{-}$ acts as a signaling molecule that regulates gene expression and many processes, including plant growth, root system architecture (Krouk et al., 2010; Alvarez et al., 2012), leaf development (Rahayu et al., 2005), seed dormancy (Alboresi et al., 2005), and flowering (Marin et al., 2011). During growth and development plants inescapably experience various forms of unfavorable environmental conditions. Under such circumstances, ${\mathrm{NO}}_{3}^{-}$ plays a key role in the processes whereby plants try to prevent any potential damage. *NRT1.5* and *NRT1.8* have been identified as two essential ${\mathrm{NO}}_{3}^{-}$ long-distance transporters (Lin et al., 2008; Li et al., 2010). Arabidopsis *NRT1.5* is expressed mainly in root pericycle cells and functions in the loading of ${\mathrm{NO}}_{3}^{-}$ into the xylem. On the other hand, Arabidopsis *NRT1.8* is expressed predominantly in xylem parenchyma cells within the vascular bundles, where it functions to remove ${\mathrm{NO}}_{3}^{-}$ from the xylem vessels. *AtNRT1.5* works together with *AtNRT1.8* to fine-tune ${\mathrm{NO}}_{3}^{-}$ long-distance transport from roots to shoots (Lin et al., 2008; Li et al., 2010). Studies showed that *NRT1.8* was strongly upregulated by Cd stress in roots, while the nrt1.8-1 mutant showed a nitrate-dependent Cd^2+^-sensitive phenotype. This finding suggests that *NRT1.8* regulated ${\mathrm{NO}}_{3}^{-}$ distribution may play an important role in Cd^2+^ tolerance in plants (Li et al., 2010). *NRT1.5* functions to mediate ${\mathrm{NO}}_{3}^{-}$ reallocation to roots, stress-responsive gene expression and metabolism; consequently salt, drought, and Cd^2+^ tolerance are affected by *NRT1.5*; further, the mRNA level of *NRT1.5* is reportedly downregulated by salt, drought, and Cd treatments; thus, lending support to the hypothesis that ${\mathrm{NO}}_{3}^{-}$ reallocation to roots might be a common response to stress, coordinately regulated by the *NRT1.8* and *NRT1.5* (Chen et al., 2012).

The plant hormone abscisic acid (ABA) regulates plant growth, seed dormancy, leaf senescence, and plant responses to abiotic forms of stress (Fujii and Zhu, 2009; Cutler et al., 2010; Gonzalez-Guzman et al., 2012; Munemasa et al., 2015; Zhao et al., 2016). Consistently, endogenous ABA level is well-known to increase under stress (Lee et al., 2006; Wang et al., 2011; Ondzighi-Assoume et al., 2016; Takahashi et al., 2018); further, it is regulated by a dynamic balance among biosynthesis, degradation, transport, conjugation, and deconjugation reactions (Finkelstein, 2013). Among conjugates, ABA glucose ester (ABA-GE) is the predominant form. ABA-GE is located in the vacuoles, in xylem sap, and probably in the cell wall (Dietz et al., 2000). BGLU10, a member of the β-glucosidase family in Arabidopsis, is localized in vacuoles, where it hydrolyzes ABA-GE to produce active ABA; this protein plays a key role in drought tolerance (Wang et al., 2011). Similarly, β-GLUCOSIDASE1 (*BGLU18*) has been shown to function in the endoplasmic reticulum (ER) to release ABA from ABA-GE in response to salt stress (Lee et al., 2006). Thus, the release of ABA from ABA-GE pools is an important mechanism for regulating ABA levels both locally and within the plant as a whole in response to stress.

Studies have shown that Cd stress triggers ethylene (ET) and jasmonic acid (JA) signaling, which converged at EIN3/EIN3-Like1 (EIL1) to modulate the expression of ethylene response factors and hence to upregulate *NRT1.8*. In contrast, ET and JA signaling mediated the downregulation of *NRT1.5* via EIN3/EIL1, and other unknown component(s). These processes enhanced stress tolerance and decreased plant growth (Zhang G.B. et al., 2014). Similarly, ABA acts as a stress response hormone; therefore, we asked, what is the relationship between ABA and *NRT1.5* and *NRT1.8* in the face of stress? We used Arabidopsis ABA mutants (*bglu10* and *bglu18*) and wild-type (Col-0) for experimental studies under Cd stress.

The available data indicate that the vacuole is involved in ion homeostasis of the cytosol by storing products of primary and secondary metabolism, and by osmoregulation, thus contributing to plant defense responses under biotic and abiotic stress. In addition, the vacuole is known to be significantly related to N use efficiency (NUE) (Andreev, 2001; Han et al., 2016; Kim et al., 2017; Liu et al., 2018; Takeda et al., 2018). Vacuolar compartmentalization of toxic or excess essential heavy metals mainly relies on tonoplast energization and the associated establishment of a proton motive-force due to the H^+^ translocating activities of V-ATPase and V-PPase and various tonoplast-localized transporters (Sharma et al., 2016). The exposure of barley seedlings to Cd led to substantially elevated transcript levels of V-ATPase subunits VHA-c and VHA-E, with the magnitude of increase being greater in the case of the latter (Finkemeier et al., 2003; Sharma et al., 2004). In a proteomic analysis of barley leaf tonoplasts, an isoform of V-PPase was observed to be upregulated by twofold during the Cd treatment (Schneider et al., 2009; Khoudi et al., 2012). As these observations indicate that V-ATPase and V-PPase seem to play an important role in the ability of plants to resist Cd, therefore, we measured V-ATPase and V-PPase activities in the Arabidopsis wild-type and in the mutants used here as experimental materials.

In both, *Brassica napus* and Arabidopsis, *NRT1.5* responded to the ABA signal by downregulating its expression under Cd stress, whereas *NRT1.8* did not respond, thus resulting in nitrate accumulation in the root to enhance its ability to resist Cd. As for Arabidopsis, the wild-type showed higher proton pump activities (V-PPase and V-ATPase), which led to less Cd being transported to the shoot, thus reducing damage caused by Cd toxicity. However, in *B. napus*, the addition of exogenous ABA directly inhibited Cd absorption by plants and enhanced their resistance to Cd toxicity.

## Results

### Arabidopsis Wild-Type (Col-0) Showed Higher Tolerance to Cd Stress Than ABA Mutants (*bglu10* and *bglu18*)

**FIGURE 1:**
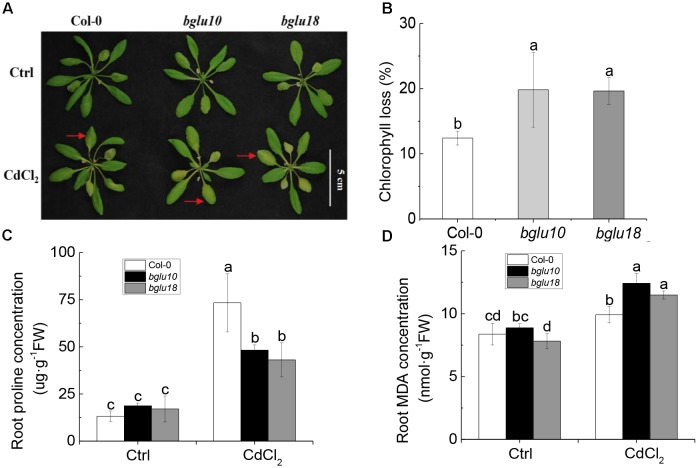
Arabidopsis abscisic acid (ABA) mutants (*bglu10* and *bglu18*) are more sensitive than wild-type (Col-0) under 20 μM cadmium stress. **(A)** Photograph showing the higher tolerance of the wild-type (Col.0) as compared with ABA mutants (*bglu10* and *bglu18*). **(B)** Chlorophyll loss in Cd^2+^ treated plants relative to control. **(C)** Effects of Cd^2+^ stress on proline in Col-0, *bglu10*, and *bglu18* plants. **(D)** Effects of Cd^2+^ stress on malondialdehyde (MDA) in Col-0, *bglu10*, and *bglu18* plants. Data represent means ± SE (*n* = 4). Bars with the same letter indicate no significant difference at *P* < 0.05 level by the method of LSD.

First, we examined the Cd phenotype by using Arabidopsis wild-type and ABA mutants (Figure 1A). There were no phenotypic differences between the two under control conditions. However, when plants were cultivated for 4 weeks under control conditions and then exposed for 3 days to 20 μM Cd, Col-0 showed more resistance to Cd, while *bglu10* and *bglu18* mutants displayed more sensitivity to Cd (Figure 1A).

Leaf chlorophyll is an important indicator of plant tolerance to Cd (DalCorso et al., 2008). We observed that after Cd stress, chlorophyll degradation rate in Col-0 was 12%, while the corresponding rates in *bglu10* and *bglu18* were both 20%, which was significantly higher than that of Col-0. This finding demonstrated that Col-0 was more tolerant to Cd than either of the ABA mutants (Figure 1B).

Proline and malondialdehyde (MDA) are also important indicators of stress tolerance. Proline was able to maintain the stability of the membrane structure and to eliminate reactive oxygen species. The accumulation of proline is positively correlated with plant stress tolerance. As for MDA, it is one of the most important products of membrane lipid peroxidation; it is cytotoxic, because it promotes cross-link polymerization of living macromolecules, such as proteins and nucleic acids. After Cd stress, proline concentration in roots of Col-0 was significantly higher than in roots of either ABA mutant. In contrast, root MDA was significantly lower in Col-0 than in ABA mutants (Figures 1C,D). Our data suggest that after Cd stress, Col-0 showed higher Cd tolerance when compared to either of the ABA mutants tested.

### Effect of Endogenous ABA on *NRT1.5* and *NRT1.8* Under Cd Stress

**FIGURE 2:**
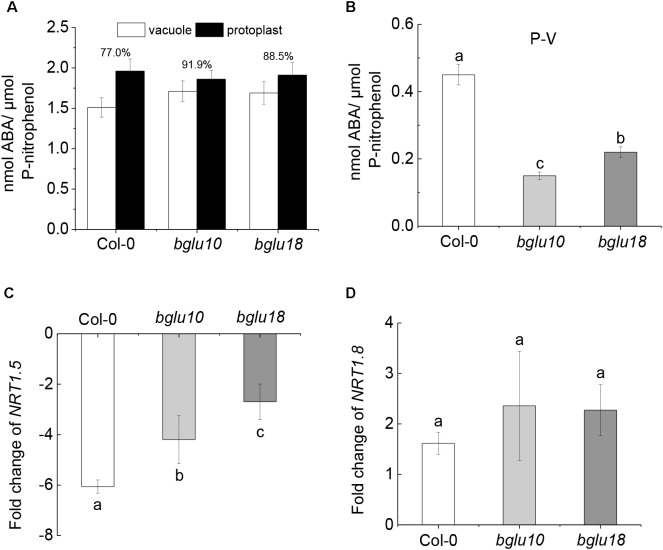
Distribution of ABA content in Arabidopsis wild-type (Col-0) and ABA mutants (*bglu10* and *bglu18*) root cells under Cd stress and the effect of endogenous ABA on *NRT1.5* and *NRT1.8*. **(A)** ABA distribution between the vacuole and protoplast; values above the bars represent the percentage of vacuolar ABA relative to the total ABA in the protoplasts. **(B)** Total ABA accumulated in cytoplasm (P-V) calculated as total ABA in the protoplast – total ABA in the vacuole. **(C)** The fold change of *NRT1.5* down-regulation in roots calculated as the expression of *NRT1.5* under normal treatment divided by the expression of *NRT1.5* under Cd stress. **(D)** The fold change of *NRT1.8* up-regulation in roots calculated as the expression of *NRT1.8* under Cd stress divided by the expression of *NRT1.8* under normal treatment. Data represent means ± SE (*n* = 4). Bars with the same letter indicate no significant difference at *P* < 0.05 level by the method of LSD.

In view of the phenotypic differences shown in Figure 1, because the materials are ABA mutants, we determined the ABA distribution and content differences in root cells under Cd stress (Figures 2A,B). We found that, compared with *bglu10* and *bglu18*, the ABA content in Col-0 root vacuoles accounted for 77.0% of protoplast ABA content, which is much lower than the ABA contents found in the ABA mutants, which were 91.9 and 88.5%, respectively (Figure 2A). Therefore, we conclude that the amount of ABA in the cytoplasm of Col-0 root cells was significantly higher than that in either the *bglu10* or the *bglu18* ABA mutant (Figure 2B).

We took the Arabidopsis roots that were grown under control conditions for 4 weeks and then treated them with 200 μM Cd for 6 h. We then tested for the gene expression of *NRT1.5* and *NRT1.8*. The expression of *NRT1.5* was significantly inhibited after Cd treatment, regardless of the material. In contrast, the expression of *NRT1.8* was significantly induced (Supplementary Figures 1a,b). However, fold change of *NRT1.5* down-regulation and *NRT1.8* up-regulation in the wild-type and the mutants was different after exposure to Cd stress. In this case, fold change of *NRT1.5* down-regulation in Col-0 was significantly higher than fold change in *bglu10* or *bglu18*. On the other hand, although the expression of *NRT1.8* was induced, there was almost no difference in fold change of *NRT1.8* up-regulation between the wild-type and the ABA mutants (Figures 2C,D). These results indicated that *NRT1.5*, but not *NRT1.8*, responded to ABA signaling.

### Effect of Proton-Pump Activity on Cd^2+^ and ${\mathrm{NO}}_{3}^{-}$ Distribution

**FIGURE 3:**
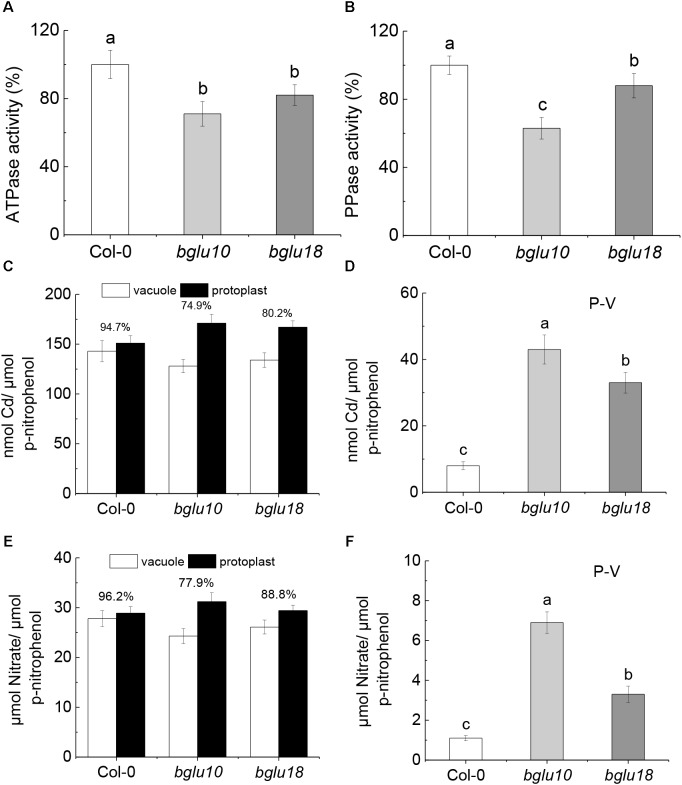
Vacuolar proton pump activity in Arabidopsis wild-type (Col-0) and ABA mutants (*bglu10* and *bglu18*) root cells under Cd stress. **(A)** V-ATPase and **(B)** V-PPase activities in roots of Arabidopsis wild-type measured and ABA mutants under treatment with 20 μmol L^-1^ CdCl_2_. The proton pumps activity of the wild-type under Cd condition was set to 100%, and the specific activity of the root tonoplast proton pumps were expressed as percentage of that in the wild-type under Cd condition. **(C)** Cd^2+^ distribution between the vacuole and protoplast in roots; values above the bars represent the percentage of vacuolar Cd^2+^ relative to the total Cd^2+^ in protoplasts; **(D)** roots total Cd^2+^ accumulated in cytoplasm (P-V) calculated as total Cd^2+^ in protoplast – total Cd^2+^ in vacuole. **(E)** ${\mathrm{NO}}_{3}^{-}$ distribution between the vacuole and protoplast in roots; values above the bars represent the percentage of vacuolar ${\mathrm{NO}}_{3}^{-}$ relative to the total ${\mathrm{NO}}_{3}^{-}$ in protoplasts; **(F)** roots total ${\mathrm{NO}}_{3}^{-}$ accumulated in cytoplasm (P-V) calculated as total ${\mathrm{NO}}_{3}^{-}$ in protoplast – total ${\mathrm{NO}}_{3}^{-}$ in vacuole. Data represent means ± SE (*n* = 4). Bars with the same letter indicate no significant difference at *P* < 0.05 level by the method of LSD.

After 3 days of 20 μM Cd treatment, there was a significant difference in proton pump activity between the wild-type and the ABA mutants tested. V-ATPase (Figure 3A) and V-PPase (Figure 3B) activities were significantly higher in Col-0 than in *bglu10* or *bglu18*. This suggests an increased ability of Col-0 plants to transport Cd^2+^ into the vacuole. The distribution of Cd^2+^ in vacuoles and protoplasts is shown in Figure 3C. As the ratio of vacuolar to protoplasmic Cd^2+^ is higher in Col-0 than that in *bglu10* or *bglu18*, the Cd^2+^ remaining in the cytoplasm in Col-0 is significantly lower than in *bglu10* or *bglu18* (Figure 3D). At the same time, the proton pump activity also influenced the distribution of ${\mathrm{NO}}_{3}^{-}$ in the cells. Additionally, the ratio of vacuolar ${\mathrm{NO}}_{3}^{-}$ to protoplasmic ${\mathrm{NO}}_{3}^{-}$ in Col-0 was higher than in *bglu10* or *bglu18* (Figure 3E), therefore, ${\mathrm{NO}}_{3}^{-}$ remaining in the cytoplasm in Col-0 was significantly lower than in *bglu10* or *bglu18* (Figure 3F).

### Higher ${\mathrm{NO}}_{3}^{-}$ and Cd^2+^ Accumulation in the Root Enhanced Stress Resistance

**FIGURE 4:**
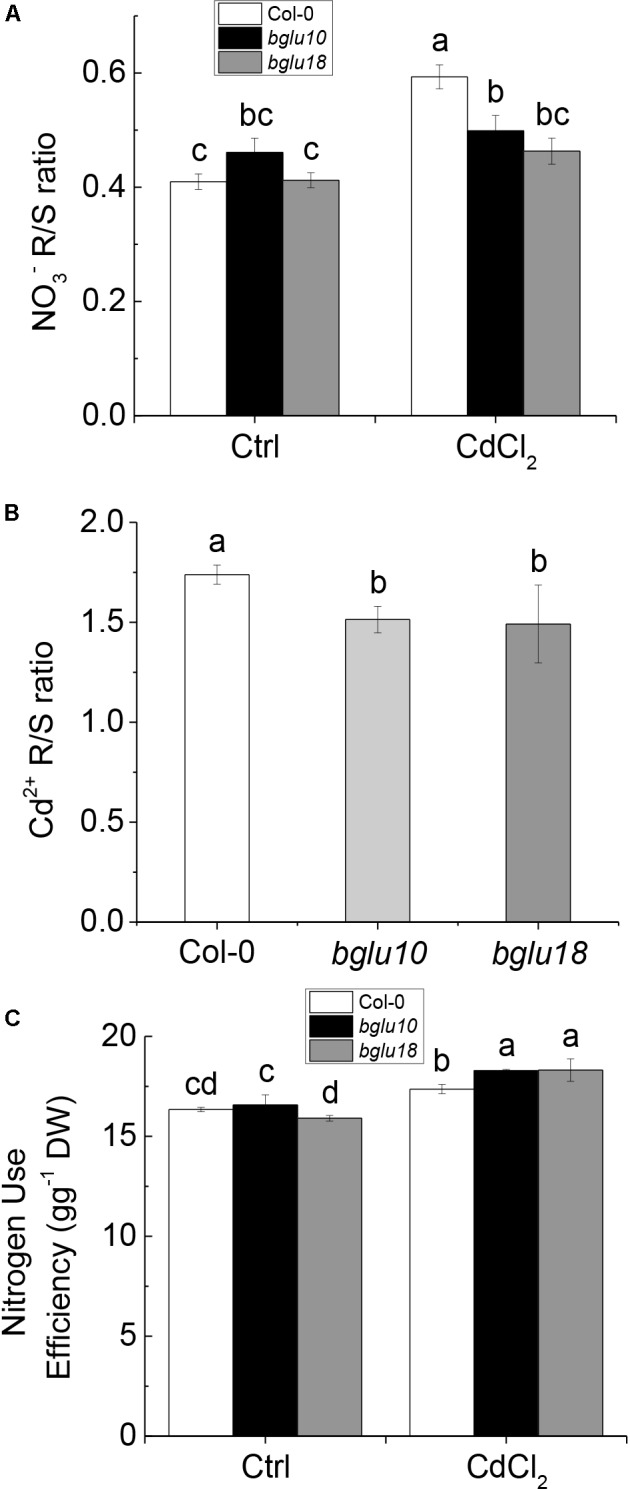
Nitrate and cadmium distribution in Arabidopsis wild-type (Col-0) and ABA mutants (*bglu10* and *bglu18*) as well as NUE (nitrogen use efficiency). **(A)** [${\mathrm{NO}}_{3}^{-}$] ration between root and shoot under control and Cd treatment. **(B)** [Cd^2+^] ration between root and shoot. **(C)** NUE of wild-type and ABA mutants under normal treatment and Cd stress. Data represent means ± SE (*n* = 4). Bars with the same letter indicate no significant difference at *P* < 0.05 level by the method of LSD.

Previous research demonstrated that stress decouples nitrate assimilation from photosynthesis through stress-initiated nitrate allocation to roots (SINAR), which is mediated by nitrate transporters *NRT1.8* and *NRT1.5*, and functions to promote stress tolerance (Li et al., 2010; Chen et al., 2012). Here, we showed that ABA produced by Arabidopsis wild-type and ABA mutants differed in response to Cd stress. The cytoplasmic ABA levels in Col-0 plants were significantly higher than those in *bglu10* or *bglu18*, which resulted in a much higher degree of inhibition of expression of *NRT1.5* in the former, whereas the level of expression of *NRT1.8* differed slightly between wild-type and mutants (Figure 2). The function of *NRT1.5* is to load the xylem nitrate into the shoot, thus, after Cd stress, Col-0 had more nitrate in the root than *bglu10* or *bglu18* (Figure 4A). Concomitantly, due to the difference in the activity of the proton pump, the amount of nitrate remaining in the cytoplasm in Col-0 was reduced, as was the nitrate transported to the shoot (Figures 3E,F). The overall result of this was more nitrate left in the roots in Col-0, thereby reflecting wild-type plant resistance to Cd.

On the other hand, due to the difference in the activity of the proton pump, the content of Cd in the cytoplasm of Col-0 was lower than in *bglu10* or *bglu18* (Figures 3C,D); thus, more Cd accumulated in the roots (Figure 4B), the net result of which was that Cd-induced damage was not as severe in Col-0 plants as in either of the ABA mutants.

In summary, the combined effects of nitrate and proton pump activity increased the resistance of Col-0 plants to Cd. Furthermore, the resistance of Col-0 to Cd was higher than that of *bglu10* or *bglu18*, but the NUE was significantly lower in Col-0 plants than in either *bglu10* or *bglu18* (Figure 4C). In order to verify the anti-Cd mechanism in plants, we treated *B. napus* with exogenous ABA and arrived at the following results.

### Exogenous ABA Enhanced Cd Resistance of *B. napus*

**FIGURE 5:**
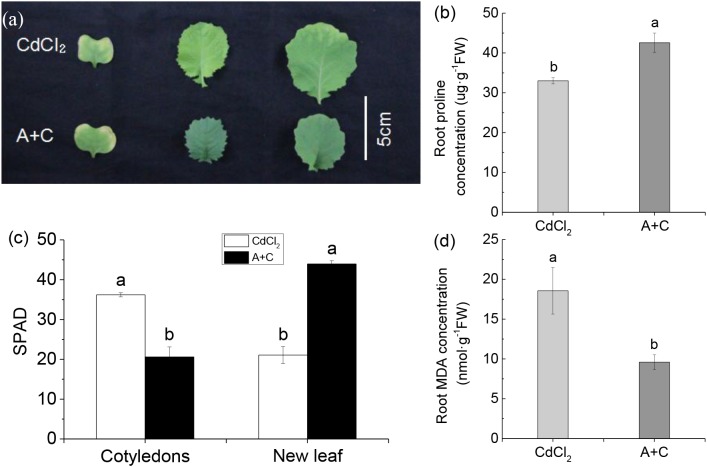
Under Cd stress, *B. napus* (814) showed stronger tolerance after adding exogenous ABA. **(a)** Photograph showing higher tolerance of the treatment with 5 μM exogenous ABA and 10 μM Cd (A+C) as compared with 10 μM Cd treatment. The leaves from left to right are cotyledons, the first new leaf and the second new leaf. **(b)** Root proline concentration in different treatments. **(c)** The SPAD of and new leaf. **(d)** Root MDA (Malonaldehyde) concentration in different treatments. Data represent means ± SE (*n* = 4). Bars with the same letter indicate no significant difference at *P* < 0.05 level by the method of LSD.

After treatment with exogenous ABA, the cotyledons of *B. napus* showed more severe yellowing than under Cd treatment alone due to the joint effects of both, ABA and Cd. ABA accelerated senescence of cotyledons, while Cd stress promoted cotyledon yellowing in. However, in this case the new leaves showed no trace of Cd poisoning, while the new leaves of *B. napus* under Cd-treatment alone showed obvious yellowing. Cd poisoning mainly affected new leaves; thus, the addition of exogenous ABA increased the anti-Cd ability of *B. napus* (Figures 5A,C). Further, after the addition of exogenous ABA, the proline concentration of *B. napus* was significantly higher than under Cd treatment alone (Figure 5B), whereas MDA concentration was significantly lower (Figure 5D). This confirmed that the addition of exogenous ABA enhanced Cd resistance of *B. napus*.

### Under Cd Stress, *BnNRT1.5* Responded to Exogenous ABA Signaling to Regulate ${\mathrm{NO}}_{3}^{-}$ Distribution, While *BnNRT1.8* Did Not Respond

**FIGURE 6:**
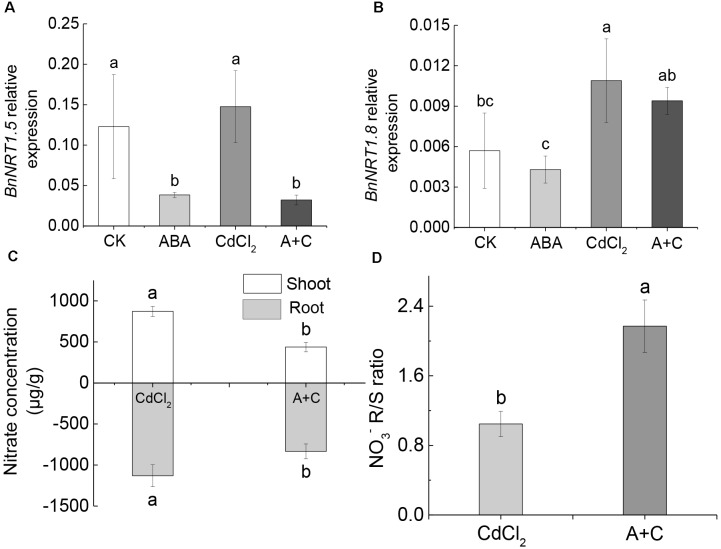
Exogenous ABA affects the distribution of ${\mathrm{NO}}_{3}^{-}$ under Cd conditions. **(A)** Gene expression of *BnNRT1.5* in different treatments (CK, ABA, CdCl_2_, A+C). **(B)** Gene expression of *BnNRT1.8*. **(C)** The concentration of ${\mathrm{NO}}_{3}^{-}$ in the shoot and root. **(D)** [${\mathrm{NO}}_{3}^{-}$] ration between root and shoot. Data represent means ± SE (*n* = 4). Bars with the same letter indicate no significant difference at *P* < 0.05 level by the method of LSD.

After the addition of exogenous ABA, the expression level of *BnNRT1.5* was significantly downregulated (sixfold), compared to Cd treatment alone (Figure 6A). However, there was no difference in the expression level of *BnNRT1.8* (Figure 6B). Under CK (normal culture) and ABA treatments, we arrived at the same conclusion: *BnNRT1.5* responded to ABA signal and the expression level was downregulated, while *BnNRT1.8* did not respond. Further, ${\mathrm{NO}}_{3}^{-}$ content in the shoots and roots under Cd treatment alone was significantly higher than in the case of Cd treatment followed by ABA addition (A+C) (Figure 6C). However, in the (A+C) treatment, the ${\mathrm{NO}}_{3}^{-}$ concentration ratio between root and shoot was significantly higher than under the Cd treatment alone (Figure 6D). This indicated that the addition of exogenous ABA caused a greater proportion of ${\mathrm{NO}}_{3}^{-}$ to be distributed in the root of *B. napus* seedlings, thereby enhancing their resistance to Cd.

### Exogenous ABA Inhibited Cd Absorption in *B. napus*

**FIGURE 7:**
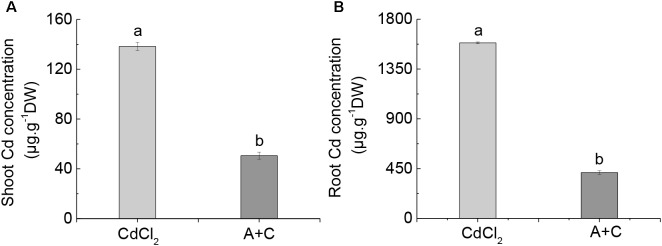
Effect of exogenous ABA on Cd uptake. Cd concentration in the shoot **(A)** and root **(B)** under Cd and (A+C) treatment. Data represent means ± SE (*n* = 4). Bars with the same letter indicate no significant difference at *P* < 0.05 level by the method of LSD.

A number of studies have reported that the addition of exogenous ABA inhibited Cd absorption and increased Cd resistance in Arabidopsis and rice (Hsu and Kao, 2003; Uraguchi et al., 2009; Fan et al., 2014). Similarly, here we observed that after the addition of exogenous ABA, the absorption of Cd was also inhibited in *B. napus*, and that shoots and roots of *B. napus* were significantly lower in Cd content than under Cd treatment alone (Figures 7A,B).

## Discussion

Based on experimental data, we demonstrated that under Cd stress, *NRT1.5* showed a response to ABA signaling, whereas *NRT1.8* showed no response, thereby resulting in nitrate accumulation in the root. Concomitantly, because of the vacuolar action of the proton pump, ${\mathrm{NO}}_{3}^{-}$ and Cd were more distributed in the vacuoles of root cells. The ${\mathrm{NO}}_{3}^{-}$ and Cd R/S ratio values showed that more ${\mathrm{NO}}_{3}^{-}$ and Cd accumulated in the roots (Figures 4A,B). These two pathways together enhanced Cd resistance in *Arabidopsis thaliana*.

Abscisic acid is known as a stress hormone that takes part in the integration of signals. ABA induces different signaling pathways to help plants resist stress. ABA induces accumulation of protectants such as small hydrophilic proteins, sugars, and proline, or activates detoxifying mechanisms that confer stress tolerance by regulating redox balance or modifying ion transport to re-establish homeostasis (Ingram and Bartels, 1996; Pompeu et al., 2017). ABA can also affect stress-induced transcription factors or some of their target-gene expression can increase stress tolerance (Peleg et al., 2011; Qin et al., 2011; Sanghera et al., 2011). We found that Col-0 was significantly more resistant to the heavy metal than either *bglu10* or *bglu18* (Figure 1). Because of the Cd stress, the levels of active ABA produced by the wild-type and the ABA mutants were different, resulting in a phenotypic difference (Figure 2). *NRT1.5* and *NRT1.8* act as long-distance transporters of ${\mathrm{NO}}_{3}^{-}$, and they respond to stress signals and act synergistically to allow more nitrate to accumulate in the root to enhance the level of plant resistance to stress (Lin et al., 2008; Li et al., 2010). The effects of *NRT1.5* and *NRT1.8* under adverse conditions are mediated by ethylene and JA (Zhang G.B. et al., 2014). In this study, we demonstrated that under Cd stress, *NRT1.5* responded to the ABA signal and the expression level was downregulated, while *NRT1.8* did not respond (Figure 2), which in turn caused more ${\mathrm{NO}}_{3}^{-}$ to accumulate in the roots (Figure 4A), thus, the Col-0 anti-cadmium ability is improved. The same conclusion was derived from experiments with *B. napus* (Figures 5, 6).

Further, V-ATPase and V-PPase play a vital role in the defense mechanisms to counter potential damage by heavy metal stress (Sharma et al., 2016). In this study, we found that Col-0, which is more resistant to Cd, showed higher V-ATPase and V-PPase activities (Figures 3A,B), which gave Col-0 a greater ability to sequester Cd in the vacuole, while a small amount of Cd remained in the cytoplasm (Figures 3C,D), caused more Cd to accumulate in the root (Figure 4B) and overall reduced the toxic effect of Cd on plants. Because there is a difference in the concentration of Cd in *B. napus* from the beginning (Figure 7), the degree of Cd toxicity in *B. napus* is different, endogenous ABA and exogenous ABA may differ in the way each counters Cd stress. Namely, the effect of endogenous ABA on the activity of the proton pump may cause accumulation of Cd in the root, while exogenous ABA seems to act by inhibiting Cd absorption to alleviate Cd toxicity. Therefore, we are not concerned about the proton pump activity in *B. napus.*

Vacuolar ${\mathrm{NO}}_{3}^{-}$ affects plant NUE (Han et al., 2016). At the same time, we found that Col-0, which showed a higher proton pump activity, accumulated more ${\mathrm{NO}}_{3}^{-}$ in the vacuoles of the roots and less ${\mathrm{NO}}_{3}^{-}$ in the cytoplasm, which resulted in less ${\mathrm{NO}}_{3}^{-}$ being transported up to the shoot (Figures 3E,F). Although the ability of the plant to resist Cd was enhanced, NUE was reduced (Figure 4C). This summarizes the roles of ${\mathrm{NO}}_{3}^{-}$ and V-ATPase and V-PPase in the improvement of Arabidopsis tolerance to Cd. This indicated a certain link between plant tolerance to stress and NUE. Indeed, generally high stress resistance would be associated with reduced NUE (Huang et al., 2018). However, it is unclear how enhanced resistance and NUE cooperate.

## Conclusion

**FIGURE 8:**
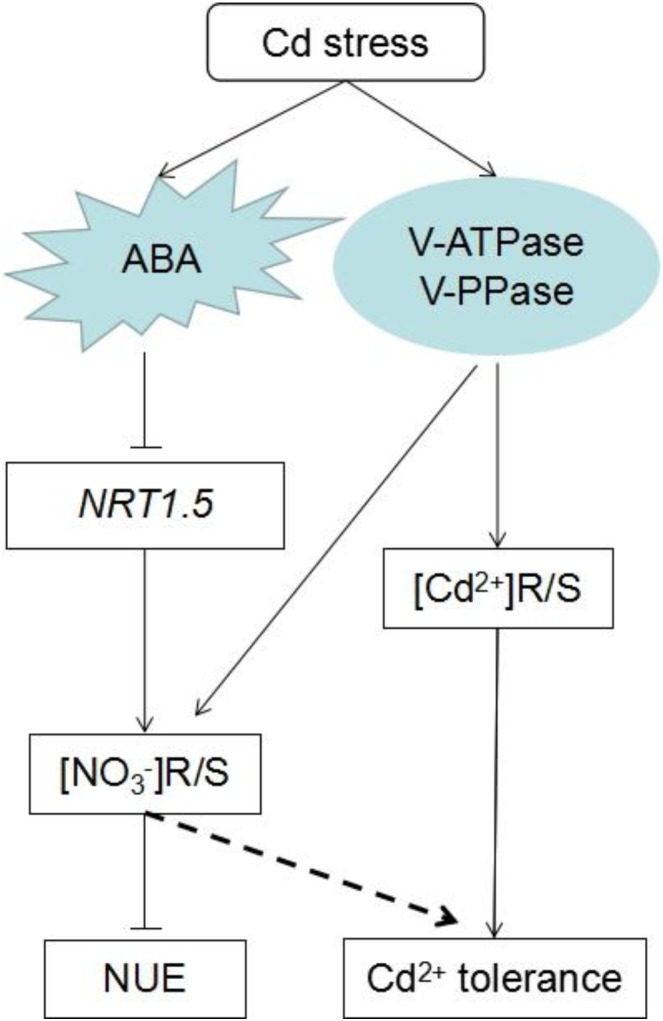
Suggested model for ABA and proton pump enhance Arabidopsis cadmium resistance.

A possible mechanism for the *NRT1.5* response to ABA signaling to trigger the accumulation of nitrate in the root and synergize with proton pump to enhance Arabidopsis resistance to Cd is schematized in Figure 8. According to this model, Cd stress induces ABA, which in turn inhibits the expression of *NRT1.5*, but has no effect on *NRT1.8*, thus causing more nitrate to be distributed in the roots; then it reduces NUE and improves Cd tolerance. Concomitantly, Cd stress enhanced the activity of the cell proton pumps in the roots, thereby causing more Cd and nitrate to be stored in the vacuole and to accumulate in the roots. More nitrate is allocated to the roots, while less Cd remains in the cytoplasm. Overall, these two processes enhance the resistance of *A. thaliana* to Cd. On the other hand, *BnNRT1.5* also responded to the ABA signal and downregulated its own expression, whereas *BnNRT1.8* showed no response. In addition, exogenous ABA hindered Cd absorption by seedlings, and then synergized with *BnNRT1.5* to enhance Cd resistance in *B. napus*.

## Materials and Methods

### Plant Material

*Arabidopsis thaliana* wild-type Columbia-0 (Col-0) was used as the control for ABA conjugate hydrolysis mutants (*bglu10* and *bglu18*). The functions of *BGLU10* and *BGLU18* have been confirmed in the reports of Wang and Lee. BGLU10, a member of the β-glucosidase family in Arabidopsis, is localized in vacuoles, where it hydrolyzes ABA-GE to produce active ABA; additionally, BGLU18 is localized in the ER, also hydrolyzing ABA-GE to produce active ABA (Lee et al., 2006; Wang et al., 2011). Mutants *bglu10* and *bglu18* used are *BGLU10* and *BGLU18* gene-deletion mutants, respectively. These were a gift from Zhang Jianhua, from the Chinese University of Hong Kong. *B. napus* (814) was provided by the Hunan Branch of Improvement Center of National Oil Crops, Hunan, China.

### Growth Conditions

Arabidopsis plants were grown in a nutrient solution in plastic pots as described in Gong et al. (2003) and Han et al. (2016). The solution was changed every 3 days, with pH adjusted to 5.8 and 0.5 g L^-1^ MES (2- (4-Morpholino) ethanesulfonic acid) was added. Pots were arranged in a completely randomized design with six biological replications. The nutrient solution consisted of 1.25 mM KNO_3_, 0.625 mM KH_2_PO_4_, 0.5 mM MgSO_4_, 0.5 mM Ca (NO_3_)_2_⋅4H_2_O, 0.025 mM Fe-EDTA, 0.25 ml L^-1^ micronutrients (stock solution concentrations were the following: 70 mM H_3_BO_3_, 14 mM MnCl_2_, 1 mM ZnSO_4_, 0.5 mM CuSO_4_, and 0.2 mM NaMoO_4_).

Soaked *B. napus* seeds were sown onto gauze fixed to an enamel pan, and soaked with deionized water. After 6-days, seedlings were transplanted into 2-L black plastic pots containing nutrient solution. The experiment was laid in a completely randomized block design with six replicates. The nutrient solution consisted of 5.0 mM KNO_3_, 1.0 mM KH_2_PO_4_, 2.0 mM MgSO_4_⋅7H_2_O, 5.0 mM Ca(NO_3_)_2_⋅4H_2_O, 0.05 mM Fe-EDTA, 9 μM MnCl_2_⋅4H_2_O, 0.8 μM ZnSO_4_⋅7H_2_O, 0.3 μM CuSO_4_⋅5H_2_O, 0.1 μM NaMoO_4_⋅2H_2_O, and 50 μM H_3_BO_3_ (Zhang D. et al., 2014). The experiments were conducted at Hunan Agricultural University in a phytotron set at 70% relative humidity, 16 h/8 h light/dark cycle (*A. thaliana*) or 14 h/10 h light/dark cycle (*B. napus*), at constant temperature (22°C). The nutrient solution for Arabidopsis plants was changed every 3-days and, after 4 weeks of cultivation, they were treated for 3-days with 20 μM Cd. The nutrient solution for *B. napus* plants was changed every 5-days and, after 10-days of cultivation, they were treated for 4-days with either 10 μM Cd or 10 μM Cd added with 5 μM ABA. *B. napus* and Arabidopsis were analyzed separately.

### Determination of Chlorophyll, Malonaldehyde (MDA), and Proline Concentrations

Leaves (approximately 0.15 g) of *A. thaliana* were sampled and extracted in 10 ml 1:1 absolute ethanol: acetone for 24 h. Absorbance was then measured at 663, 645, and 652 nm to determine chlorophyll concentration. Chlorophyll loss (a) was calculated as the chlorophyll concentration under the control conditions (b) minus chlorophyll concentration under Cd stress, and (c) divided by concentration under control conditions, i.e., a = (b-c)/c^∗^100. MDA and proline were measured in root tissues. For MDA, 0.5 g of root tissue was ground in 5 ml 5% TCA, then centrifuged at 925 × *g* for 10 min. The supernatant was collected and used for determination of MDA concentration using the modified thiobarbituric acid–malondialdehyde (TBA–MDA) assay (Song et al., 2014). Proline was assayed according to the method described in Bates et al. (1973) and Sharma and Dubey (2005). Briefly, root tissues (0.5 g) were sampled and ground in 5 ml of 3% sulfosalicylic acid, then centrifuged at 22000 × *g* for 5 min. The supernatant was collected and used for determination of proline concentration by reaction with acidic ninhydrin (Chen et al., 2012).

### Determination of ${\mathrm{NO}}_{3}^{-}$ and Cd^2+^ Concentrations in Intact Protoplasts and Vacuoles

Root tissues of *A. thaliana* (0.5 g) were collected to isolate intact protoplasts and vacuoles as described in Robert et al. (2007), with minor modifications as outlined in Huang et al. (2012) and Han et al. (2016). Purified protoplasts and vacuoles were subsampled and used to determine ${\mathrm{NO}}_{3}^{-}$ and Cd^2+^ concentrations (Vögeli-Lange and Wagner, 1990) and for enzyme activity assays (Ma et al., 2005). ${\mathrm{NO}}_{3}^{-}$ concentration in protoplasts and vacuoles were measured by a continuous flow auto-analyzer (Auto Analyzer 3, Bran and Luebbe, Norderstedt, Germany) as described previously (Han et al., 2016). The activities of acid phosphatase (ACP) and cytochrome oxidase (COX) were determined using plant ACP colorimetry and COX assay kits (GenMedSci, Inc., Shanghai, China) following the instructions by the manufacturer. ACP activity specific to vacuoles was determined and used to normalize ${\mathrm{NO}}_{3}^{-}$ accumulation. We measured ${\mathrm{NO}}_{3}^{-}$ in the protoplast outside the vacuole, which includes the cytosol and organelles, e.g., mitochondria and Golgi Apparatus (Robert et al., 2007). As most ${\mathrm{NO}}_{3}^{-}$ in the protoplast outside the vacuole is located in the cytosol (Krebs et al., 2010), we refer to ${\mathrm{NO}}_{3}^{-}$ distribution between vacuoles and cytosol rather than vacuole versus protoplast; Cd^2+^ concentrations in protoplasts and vacuoles were measured by inductively coupled plasma-mass spectrometry (ICP-MS, ELAN DRC-e, PerkinElmer, Shelton, United States) as described in Huang et al. (2012), with the corresponding modification.

### Determination of V-ATPase and V-PPase Activities

V-ATPase and V-PPase activities within microsomal membranes collected from the root tissues of *A*. *thaliana* were colorimetrically determined as Pi release after an incubation period of 40 min at 28°C (Zhu et al., 2001; Krebs et al., 2010; Han et al., 2015). Reactions were terminated by adding 40 mM citric acid. For the blank value, 10 μg of bovine serum albumin was used instead of tonoplast vesicles. The V-ATPase assay medium contained 25 mM Tris-MES (pH 7.0), 4 mM MgSO_4_⋅7H_2_O, 50 mM KCl, 1 mM NaN_3_, 0.1 mM Na_2_MoO_4_, 0.1% Brij 35, 500 μM NaVO_4_, and 2 mM Mg-ATP. Activity was expressed as the difference in Pi release measured in the absence and in the presence of 100 nM concanamycin A. V-PPase was assayed in a reaction medium containing 25 mM Tris-MES (pH 7.5), 2 mM MgSO_4_ × 7H_2_O, 0.1 mM Na_2_MoO_4_, 0.1% Brij 58, and 0.2 mM K_4_P_2_O_7_. V-PPase activity was calculated as the difference in Pi release measured in the absence and the presence of 50 mM KCl.

### Determination of ${\mathrm{NO}}_{3}^{-}$ Concentration

Nitrate was extracted from tissue samples (shoot: 1 g; root: 0.5 g) in deionized water for 30 min in a boiling water bath; next, 0.1 ml of the sample solution was taken, 0.4 ml of 5% salicylic acid-sulfuric acid solution was added, and mixed. After cooling, the mixture was cooled at room temperature for 20 min, and then 9.5 ml of an 8% sodium hydroxide solution was added. The sample was then allowed to cool to room temperature and spectrophotometrically determined for nitrate at 410 nm (Cataldo et al., 1975).

### Determination of Biomass, N and Cd^2+^ Concentrations

Whole, hydroponically grown seedlings of *B. napus* and *A. thaliana* were sampled, oven-dried to constant weight, first at 105°C for 30 min, followed by 70°C. N concentration was determined as described by Han et al. (2016) (N data is used to calculate NUE). For the Cd^2+^ assay, shoots and roots were sampled separately, dried, and weighed; Cd^2+^ concentration was then determined by ICP-MS, after digesting with 4:1 HNO_3_: HClO_4_ (Huang et al., 2012).

### Determination of ABA Concentration

Endogenous ABA was extracted from the isolated vacuoles and protoplasts of each sample using 0.5 mL of homogenizing buffer (70% methanol, 0.1% formic acid); 2 ng ABA-d6 (Olchemim, Olomouc, Czechia) were added to the extracts as an internal standard (Balcke et al., 2012). The mixture was diluted twice using deionized water, and the ABA concentration of a 50-μL dilution of each sample was determined using the UPLC-TripleTOF 5600+ system (Sciex, Concord, ON, Canada).

### Real-Time Reverse Transcription-PCR Analysis

Root samples were ground in liquid nitrogen. Total RNA was extracted with TRIzol (Ambion, United States). The first-strand cDNA was synthesized using the total RNA by PrimeScript reverse transcription (RT) reagent kit (TaKaRa, Shiga, Japan). The qRT-PCR assays for the detection of relative gene expression were performed using SYBR^®^ Premix Ex TaqTM II (Tli RNaseH Plus) (TaKaRa, Shiga, Japan) with an Applied Biosystems StepOneTM Plus Real-time PCR System (Thermo Fisher Scientific, Waltham, MA, United States). The thermal cycles were as follows: 95°C for 3 min, followed by 40 cycles of 95°C for 10 s, then 60°C for 30 s. Melt curve analysis to ensure the primer gene-specificity was conducted as follows: 95°C for 15 s, 60°C for 1 min, 60–95°C for 15 s (+0.3°C per cycle). The gene-specific primers for qRT-PCR assays are listed in Supplementary Table 1 (Bustin et al., 2009; Wang et al., 2014).

### Statistical Analysis

We used the SPSS software (IBM SPSS Statistic 19) for ANOVA and mean separation of main effects and interactions using LSD’s multiple range test at *P* < 0.05. Data are means and SE of three or six replicates from three independent experiments. Different letters associated with specific data (e.g., at the top of histogram bars in figures) indicate significant differences at *P* < 0.05.

## Author Contributions

TW and ZZ designed the experiments and all co-authors wrote the manuscript. TW performed most of the experiments. TW, YH, and ZZ analyzed the data.

## Conflict of Interest Statement

The authors declare that the research was conducted in the absence of any commercial or financial relationships that could be construed as a potential conflict of interest.
