# Supplementary material for: Mechanism Enhancing Arabidopsis Resistance to Cadmium: The Role of NRT1.5 and Proton Pump
Source: Front Plant Sci. 2018 Dec 19;9:1892. doi: 10.3389/fpls.2018.01892 (PMC6305759; doi:10.3389/fpls.2018.01892)
Supplement: Supplementary file 1 [file Data_Sheet_1.PDF]

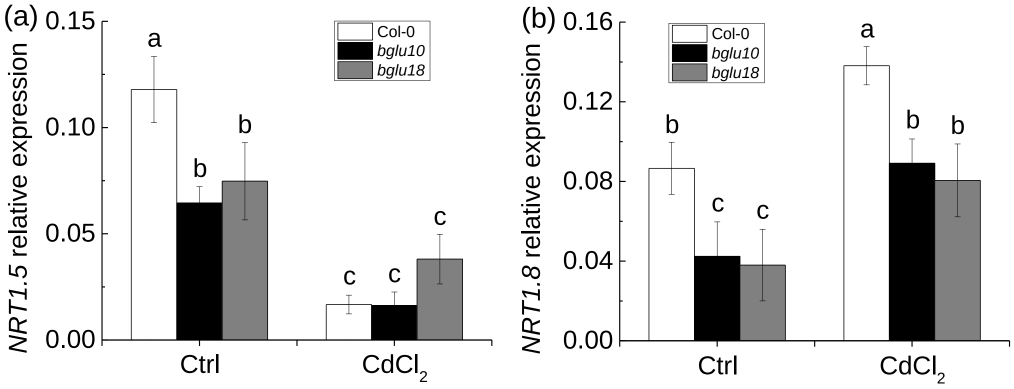

Supplementary Figure 1 Gene expression of *NRT1.5* and *NRT1.8* in roots under normal culture and Cd treatment. (a) Gene expression of *NRT1.5*. (b) Gene expression of *NRT1.8*. After 28 days of normal culture, 20 μM Cd was treated for three days. Data represent means ± SE (n=4). Bars with the same letter indicate no significant difference at P<0.05 level by the method of LSD.

Supplemental Table 1 Gene-specific primers used for qRT-PCR assays in this study

Gene name	Forward (5´-3´)	Reverse (5´-3´)
AtNRT1.5	TGGAGCGTTTCTCAGCGATT	TCCATCATGGAATGTGAACCAC
AtNRT1.8	TCTTCATCTTCGCATACAGGCGGT	GCCATTATCGCAATCACAAGCCCA
SAND	GATGAGGATGATGCTTCTACG	CCTGAGCGTTGTATCTTGGT
BnNRT1.5	GATGAAGTCACGCCTTGCG	GCAATGTTCGGTTGGTAACCC
BnNRT1.8	GGGTATGGTGGTTATCAGCCC	CGAAAGGAGCGATCCGAGG
BnACT7	GCTGACCGTATGAGCAAAG	AAGATGGATGGACCCGAC
BnEF1α	GCCTGGTATGGTTGTGACCT	GAAGTTAGCAGCACCCTTGG
BnGDI1	GAGTCCCTTGCTCGTTTCC	TGGCAGTCTCTCCCTCAGAT
